# The Administration of Ovarian and Other Extracts

**Published:** 1919-11-08

**Authors:** 


### SOME ASPECTS OF ENDOCRINOLOGY.

### The Administration of Ovarian and other Extracts.

Like so many of the more recent medico-scientific advances, the therapeutic aspect of our discoveries regarding the endocrine glands has suffered considerably from deficient correlation between laboratory and clinical work. The subject is at the moment at its most interesting chapter, immense possibilities appear on every hand, and from it may be expected to develop some of the most valuable additions to medical knowledge in our time. Thus we are indebted to Dr. A. W. Lescohier for some concise comments on the present state of endocrinology as recently published in the American *Medical Record.* He writes essentially from the practical point of view and his illustrations of the correct applications of glandular treatment are well worth the physician's attention.

### Empiricism.

The physiologist has already so definitely established the relations between the various members of the endocrine group and both the normal and pathological states that it has for some time been possible to consider many important symptom complexes in terms of internal secretory disorders; but the exact methods which have led up to these conclusions have unfortunately not been applied to the therapeutic applications. Empiricism, rather than scientific study, has been, and still is, largely the outstanding factor, and nothing could be more fatal to the success of so promising a form of treatment. Two erroneous views towards the subject have been taken by medical men. There is the, perhaps, too large proportion of practitioners who are ready to embrace the first hypothesis put forward, and who rush to apply it to their own practice; and opposed to them are the sceptics who frankly question the value of glandular therapeutics except on the most clear-cut indications. To both the author explains that, "while deprecating the extravagant ideas of the unbalanced enthusiast, it must be conceded, on the other hand, that there is an unwarranted tincture of pessimism in certain of the criticisms which have been levelled at glandular therapy. There are positive as well as negative facts to be remembered, and the real usefulness of our knowledge of its secreting glands is largely to be measured by the degree in which it can be utilised in treatment. The crying need of endocrinology at present is carefully controlled by therapeutic investigation."

### By Way of Illustration.

At present the physician trained to appreciate the minor endocrine aberrations is distinctly the exception. We have long known the signs and symptoms of myxœdema and cretinism and their diagnosis is by no means difficult. The results of their treatment are definite, but illustrations we have ourselves given* in this journal of other smaller manifestations of thyroid abnormalities serve to show how, although instances are relatively common, yet the doctor must be specially taught to recognise these things. The disturbances of the female generative system, attributable to endocrine aberration, form a further definite class, and here again the relations between ovarian activity and the menstrual function have long been recognised, while more recently there have been established connections between the thyroid, pituitary, and mammary glands, and the development and functional activities of the sexual organs. Much physiological discussion has already raged round many of these matters, but the author sets out merely to emphasise a few main points important from the therapeutic aspect.

### Ovarian Activity.

The results of administration of ovarian or corpus luteum extract in controlling the disturbances at the menopause are not so consistent as might be expected, and to act correctly we must realise that "the failure in some of these cases is undoubtedly to be attributed to the complicating glandular factors; in other words, the disturbances of the menopause, whether occurring physiologically or as the result of removal or destruction of the ovaries, are to be interpreted as a general disturbance of the *endocrine balance,* in which the dominating factor is subject to variation." It is this *balance* we must correct. More definite evidence appears in connection with disorders of menstruation, and we agree with the experience that, although there is again no perfect parallel between either glandular deficiency or hyper-activity and the intensity of the menstrual function, yet there is considerable proof that a very definite group of cases benefit by ovarian therapy—namely, those of the true deficiency type. But careful diagnosis is needed. They include patients, physically under-developed, those whose menses from puberty have been irregular or scanty; those in whom the menstrual function has been normal but subsequently becomes irregular or deficient; and cases of amenorrhœa associated with hysterical temperament. In all these, with the exception of the last, where care must be taken not to attribute failure to glandular therapy when applied to a person with a nervous system of inherent instability, experience is greatly in favour of the efficacy of ovarian preparations. But remember that, "On the other hand, there is no doubt that very profound nervous disturbances and occasionally psychoses may result from ovarian insufficiency during the active sex period of life."

Dr. Lescohier draws attention to a point not always appreciated in connection with dysmenorrhœa. In spite of cures being reported as

* "Minor Thyroid Insufficiency," The Hospital, May 10, 1919.

**Some Aspects of Endocrinology—(*continued*).** following ovarian administration, it is obvious that we should expect a hyperfunction of the ovaries rather than a deficiency to occur in this connection, and that ovarian treatment would be strongly contraindicated.

Possibly some cases of dysmenorrhœa may be due to a hypersensitive nervous system of which ovarian deficiency is a predisposing cause, and in these administration is correct, but the summary of our present knowledge probably amounts to the fact "that the usefulness of such treatment is largely to be limited to the early pubertal period as an agent to promote the development of the internal genitalia."

Antagonistic Glandular Action.

Another good instance is provided. A certain group of cases of menorrhagia may be associated with excessive ovarian activity. Therefore is ovarian extract administration completely contra-indicated, but it is not a little interesting that in these instances preparations from the mammary gland appear to be of great value and to have a directly neutralising effect. "The abatement of the menses during gestation and the rarity of impregnation during the period of lactation are illustrations of the relation existing between these two glands. The therapeutic side of the question has not been exhaustively studied, but such clinical evidence as is available indicates that the mammary exerts a definite effect in neutralising ovarian hypersecretion."

In Conclusion.

As the author explains, he gives merely passing references and illustrative examples to prove that endocrine therapy offers treatment possibilities of the greatest importance, but which will never materialise until empirical methods of administration are abandoned. The glandular syndromes must be realised and appreciated before any successful attempt can be made correctly to develop this new medical chapter. Obviously, so great a matter can be but briefly sketched in a single article, but the points made should serve to make at least some of those who work on the "cut-and-try method" realise the futility and often dangerous nature of their efforts.

